# Detection of cognitive impairment, dementia and associated risk factors among Aboriginal and Torres Strait Islander peoples: Retrospective baseline audit results from a stepped‐wedge cluster‐randomised controlled trial

**DOI:** 10.1111/ajag.70007

**Published:** 2025-03-05

**Authors:** Kate Bradley, Jo‐anne Hughson, Zoë Hyde, David Atkinson, Sarah Russell, Rachel Quigley, Dawn Bessarab, Leon Flicker, Kylie Radford, Kate Smith, Edward Strivens, Sandra Thompson, Irene Blackberry, Mary Belfrage, Robyn Smith, Roslyn Malay, Belinda Ducker, Kylie Sullivan, Wendy Allan, Bonnie Giles, Tina Humphry, Louise M. Lavrencic, Dimity Pond, Dina LoGiudice

**Affiliations:** ^1^ The University of Melbourne, Faculty of Medicine, Dentistry and Health Sciences, Royal Melbourne Hospital Melbourne Victoria Australia; ^2^ Western Australian Centre for Health and Ageing, Medical School University of Western Australia Perth Western Australia Australia; ^3^ Rural Clinical School of Western Australia The University of Western Australia Broome Western Australia Australia; ^4^ College of Medicine & Dentistry James Cook University Cairns Queensland Australia; ^5^ Centre for Aboriginal Medical and Dental Health, Medical School University of Western Australia Perth Western Australia Australia; ^6^ Neuroscience Research Australia Sydney New South Wales Australia; ^7^ Western Australian Centre for Rural Health The University of Western Australia Geraldton Western Australia Australia; ^8^ John Richards Centre for Rural Ageing Research La Trobe University Wodonga Victoria Australia; ^9^ Derbarl Yerrigan Health Service Perth Western Australia Australia; ^10^ Berowra Family Medical Practice Sydney New South Wales Australia

**Keywords:** ageing, Australian Aboriginal and Torres Strait Islander peoples, dementia, risk factors

## Abstract

**Objective:**

Aboriginal and Torres Strait Islander peoples experience high rates of dementia, cognitive impairment not dementia (CIND) and associated risk factors. The objective of this paper is to outline baseline audit results of documented dementia, CIND and associated risk factors in patients attending Aboriginal Community‐Controlled Health Organisations (ACCHOs).

**Methods:**

Twelve ACCHOs in urban, regional and remote locations across Queensland, New South Wales, Victoria and Western Australia participated in the study. A specialised audit tool identified documented CIND, dementia and risk factors. Medical record audits of 1655 clients aged 50 years or older for the period from 1 September 2016 to 31 January 2019 were completed.

**Results:**

The mean age of patients was 60.3 ± 8.2 years, and 57% were female. The overall prevalence of documented CIND or dementia was low, noted for only 67 (4%) patients. The prevalence of risk factors was high, with over two thirds (71%, *n* = 1168) of the cohort having ≥4 risk factors associated with dementia and CIND. These included high rates of hypertension (56%), diabetes (45%), dyslipidaemia (48%), obesity (40%) and current smoking (42%).

**Conclusions:**

There was a low detection of CIND and dementia accompanied by a high prevalence of associated risk factors in this primary health‐care setting. These findings highlight the need to improve dementia and CIND detection in Aboriginal and Torres Strait Islander patient groups across varied geographical settings. The findings also provide insights into risk factor prevalence to inform management strategies. Responsive models of cognitive care that are culturally appropriate and co‐designed with ACCHOs are required to address this need.

**Practice impact**

This audit of documented dementia, CIND and associated risk factors in ACCHOs demonstrates high rates of risk factors and low detection of CIND and dementia. These results highlight a need for improved culturally responsive dementia prevention strategies in primary health‐care settings for Aboriginal and Torres Strait Islander peoples.

## 1 INTRODUCTION

Aboriginal and Torres Strait Islander peoples are the first Australians and are the keepers of the longest continuing cultures in the world, having lived in Australia for more than 60,000 years.^1^ There are many examples of Aboriginal and Torres Strait Islander people ageing well, and population projections indicate exponential growth in the older population by mid‐century.^2^ Yet, Aboriginal and Torres Strait Islander communities, like other colonised populations, experience a health status below that of the non‐Indigenous population, including high rates of cognitive impairment and dementia.^3^, ^4^, ^5^, ^6^ Previous work also shows that onset occurs at a younger age.^3^, ^4^, ^5^

Prevention and management of vascular and other risk factors may reduce dementia risk.^7^, ^8^, ^9^ This is relevant in Aboriginal and Torres Strait Islander populations where there is a high incidence of comorbidity occurring at an earlier age,^10^, ^11^, ^12^ including potentially modifiable risk factors, such as polypharmacy^13^ and chronic diseases.^11^ The three cohort studies that have been undertaken in diverse regions of Australia identified a range of potentially modifiable risk factors. For example, in remote Aboriginal communities in Western Australia, head injury was found to be a significant longitudinal risk factor for cognitive decline, and cross‐sectional associations included smoking, stroke, poor mobility, incontinence and falls.^14^ A study of Aboriginal communities in regional and urban New South Wales (NSW) found childhood adversity and psychological trauma, a history of unskilled work, stroke and head injury to be independent predictors of dementia. Other associated dementia risk factors included social isolation and depression.^15^, ^16^ In the Torres Strait, kidney disease and cerebrovascular disease were significantly associated with dementia.^17^

Primary health‐care organisations play a central role in the detection and ongoing management of dementia in the community. They are well positioned to support programs designed to increase detection of cognitive impairment and dementia and to address potentially modifiable dementia risk factors. However, rates of dementia detection within primary health‐care settings are generally low.^18^ Factors resulting in poor detection of dementia and CIND in these settings include lack of confidence to diagnose, therapeutic nihilism, lack of access to specialist services, time constraints, poor follow‐up of abnormal findings and high staff turnover.^18^, ^19^ Prior research by our team in the Kimberley region of Western Australia found that only 38% of those with dementia and 3% with CIND had been diagnosed by general practitioners (GPs) or had their diagnoses documented.^20^

The National Aboriginal Community Controlled Health Organisation's (NACCHO) Continuous Quality Improvement (CQI) Framework encourages the use of CQI frameworks to help build capability within Aboriginal primary healthcare to support the delivery of high‐quality, responsive and culturally safe health services that meet the specific needs of Aboriginal and Torres Strait Islander peoples.^21^ Aligning with the recommendations of the NACCHO‐CQI framework, a medical record audit tool was developed to collect data on dementia risk, detection and management and help identify where improvements in practice could be made. The aim of this paper is to report baseline audit results on the rates of dementia, CIND and dementia risk factors documented for patients attending ACCHOs.

## 2 METHODS

### 2.1 Trial design

The baseline audits described in this paper were conducted retrospectively on medical records for the period from September 2016 to January 2019 (inclusive). The audits were conducted as part of a stepped‐wedge cluster‐randomised controlled trial focusing on the implementation of a culturally responsive best‐practice model of dementia care for 12 ACCHOs across four states. Justification and in‐depth explanation of the trial design have been published in the study protocol.^20^

### 2.2 Materials

Baseline audits were conducted by trained research staff who accessed a sample of client medical records in each ACCHO to capture patients' dementia risk profile, evidence of assessment, investigations or follow‐up relating to cognition, as well as documentation of the diagnosis of dementia or CIND over the preceding 2 years. The audit tool is reproduced in the accompanying Appendix S1. To maximise inter‐rater reliability across sites, research assistants were trained to use a standardised procedure, and a sample of audits was checked by independent auditors at each site. De‐identified data were entered into a secure web‐based data collection support system (REDCap™). Data were checked and cleaned prior to analysis.

### 2.3 Outcome measures

In this study, terms used to describe neurocognitive disorders were reflective of descriptors documented by health professionals in medical records we observed when testing and refining the audit tool. Mild cognitive impairment (MCI) was a diagnostic term commonly found in the audits. MCI is a widely accepted term referring to cognitive impairment that sits between normal ageing and dementia with minimal impairment of instrumental activities of daily living.^22^ Cognitive impairment associated with other diagnoses—for example, depression, delirium and head trauma—and identified cognitive impairment without a confirmed diagnosis were also recorded during auditing. We have used the term ‘cognitive impairment not dementia’ (CIND) to encompass all of these terms.

The *co*‐*primary outcome measures* of the baseline audits are documentation of dementia or CIND in the medical health records and management of dementia or CIND with regard to diagnostic activities, including (i) evidence of cognitive assessment and/or (ii) documentation of inquiry into cognitive health (e.g. asking questions regarding memory; obtaining collateral/carer report regarding cognition); (iii) laboratory or imaging investigations specifically requested for assessment of dementia; and (iv) referral to a memory clinic/geriatrician. Documentation of dementia risk factors was also recorded to inform management of dementia throughout the study, and baseline prevalence is described in this paper. Included risk factors were those generally found to be recorded in patient medical records when the audit tool was trialled (see the Audit Tool in Appendix S1).

### 2.4 Participants

Twelve co‐researching partner ACCHOs were involved in the study; three from each of the following states: New South Wales, Queensland, Victoria and Western Australia. The study partners were recruited through existing good relationships with the practices built during previous research and collaborative projects. The ACCHOs were initially engaged through discussions between senior management staff and project investigators. Medical records from a cohort of patients were audited in each ACCHO. Audit inclusion criteria were: (i) Aboriginal and/or Torres Strait Islander persons aged 50 years or older; (ii) active patients of the ACCHO (using the Royal Australian College of General Practitioners' [RACGP] criteria)^23^; (iii) not seriously ill (expected to be alive 6 months from audit start date); (iv) habitually residing in the local area (also including transient community members); and (v) not residing in residential aged care at the outset of the study.

### 2.5 Consent

The project protocol was provided to the senior management at all ACCHOs, and consents and/or letters of support were obtained for participation in the study from each partnering organisation.^20^ Participating ACCHOs authorised the use of routine clinical data for this research project under strict conditions of confidentiality and deidentification. Ethics approvals obtained for this study allowed for a waiver of individual informed consent for the audit data collection. No minors were involved in the study.

### 2.6 Sample size

Sample size calculations, detailed in the published study protocol, indicated that 660 people were required for analyses.^20^ To allow for attrition, we aimed to recruit 150 people from each ACCHO. For ACCHOs with ≤150 patients meeting eligibility criteria, all records at that ACCHO were audited. For ACCHOs with >150 eligible patients, every *n*th person was selected from an alphabetical list of patients, where *n* was the total group size divided by the size of the desired sample (*n* = 150). For example, every fourth person would be audited at a clinic with 600 eligible patients. In total, medical records of 1660 people were audited (between 79 and 156 patients at each ACCHO). Five patients were subsequently found to be aged less than 50 years and were excluded from analyses.

### 2.7 Statistical analyses

Data were analysed with Stata SE, version 17 (StataCorp, College Station, Texas). Sociodemographic and clinical data for patients are presented as means and standard deviations for continuous variables, and as proportions for categorical variables. Differences between groups of patients were explored using ordinary least‐squares linear regression with cluster‐robust standard errors and *χ* ^2^ tests, adjusted for clustering as appropriate, and *p*‐values less than .05 were considered statistically significant.

Ethics approval was obtained from the Aboriginal Health and Medical Research Council Human Research Ethics Committee (reference numbers: 1362/18 and 1855/21), The University of Melbourne Human Research Ethics Committee (IDs 1851943 and 12140), the Western Australian Aboriginal Health Ethics Committee (reference number 858) and James Cook University HREC (Ref H7371). The study was supported by the Kimberley Aboriginal Health Planning Forum (Ref 2018–006). A waiver of the requirement for individual informed consent was provided by the research ethics committees that approved this study as audit data collection was deemed to align with existing quality assurance processes and informed organisational consent and/or letters of support were provided by all participating ACCHOs.

## 3 RESULTS

Demographic and clinical characteristics of the 1655 patients included in this study are outlined in Table 1. The mean age was 60.3 ± 8.2 years, and 57% were female. Cognitive impairment was documented for 67 patients (4%); 29 (2%) had a diagnosis of dementia, and 38 (2%) had CIND. Subtypes of dementia and CIND are shown in Table 1.

**TABLE 1:** Demographic and clinical characteristics of patients.

Characteristic	*n* (%) or mean ± SD
Age (years)	60.3 ± 8.2
Identify as	
Aboriginal	1533 (93)
Torres Strait Islander	74 (5)
Aboriginal and Torres Strait Islander	48 (3)
Sex	
Male	720 (44)
Female	935 (57)
Cognitive impairment identified	67 (4)
Total dementia diagnoses	29 (2)
Alzheimer's disease	13 (1)
Vascular dementia	5 (0)
Mixed dementia	5 (0)
Lewy body dementia	0 (0)
Parkinson's dementia	1 (0)
Frontotemporal dementia	1 (0)
Young‐onset dementia	1 (0)
Dementia not otherwise specified	8 (1)
Total with CIND	38 (2)
Mild cognitive impairment	11 (1)
Depression	4 (0)
Delirium	4 (0)
Medication‐related	0 (0)
Head trauma	2 (0)
No confirmed diagnosis	9 (1)
Other diagnosis	15 (1)
Any evidence of assessment of cognition	519 (31)
Assessment of cognition with tools	209 (13)
Mini Mental State Examination (MMSE)	187 (11)
Kimberley Indigenous Cognitive Assessment (KICA)	13 (1)
Clock‐drawing test	11 (1)
General Practitioner Assessment of Cognition (GPCOG)	1 (0)
Rowland Universal Dementia Assessment Scale (RUDAS)	6 (0)
Other tool(s)	15 (1)

*Note*: The dementia, CIND and assessment tool sub‐categories are not mutually exclusive. The CIND subtype *other diagnosis* refers to any diagnosis different from mild cognitive impairment, depression, delirium, medication‐related and head trauma, but not including dementia. The *no confirmed diagnosis* category refers to situations where cognitive impairment was identified but no evidence of a formal diagnosis was available in the medical record.

Abbreviation: CIND, cognitive impairment not dementia.

**FIGURE 1:**
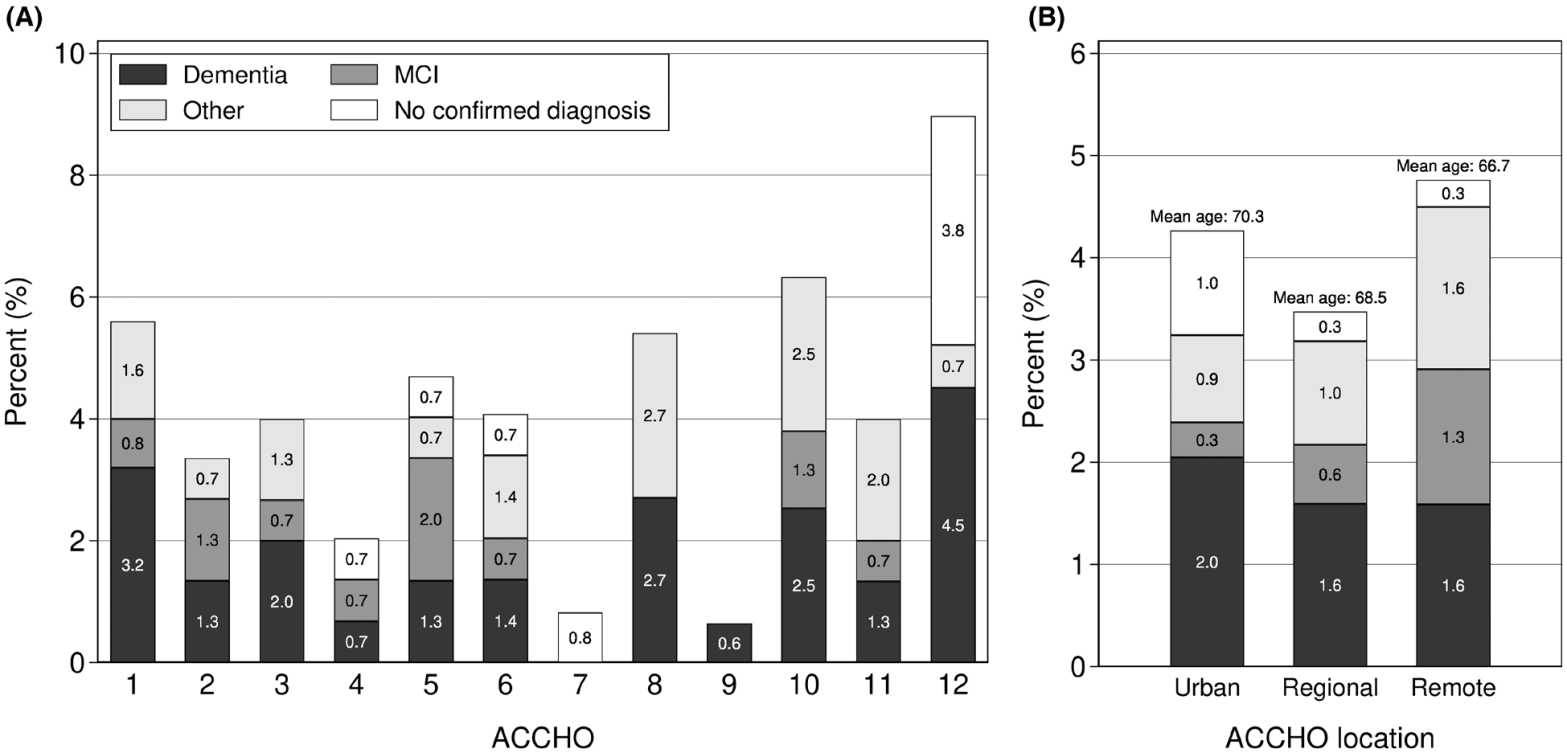
Proportion of patients with cognitive impairment by Aboriginal Community‐Controlled Health Organisations (ACCHO) (A) and ACCHO location (B). Ages shown in Panel B are for patients diagnosed with cognitive impairment. MCI, mild cognitive impairment. Cognitive impairment not dementia (CIND) comprises the categories MCI, other diagnoses and no diagnosis.

The prevalence of documented cognitive impairment varied markedly between ACCHOs, ranging between 1% and 9% (Figure 1A). However, the mean age of all patients at the 12 ACCHOs also varied, ranging from 58.7 ± 7.4 to 62.1 ± 8.7 years (*p* < .001). As shown in Figure 1B, the prevalence of documented cognitive impairment was broadly similar in urban, regional and remote locations (*p =* .74).

**FIGURE 2:**
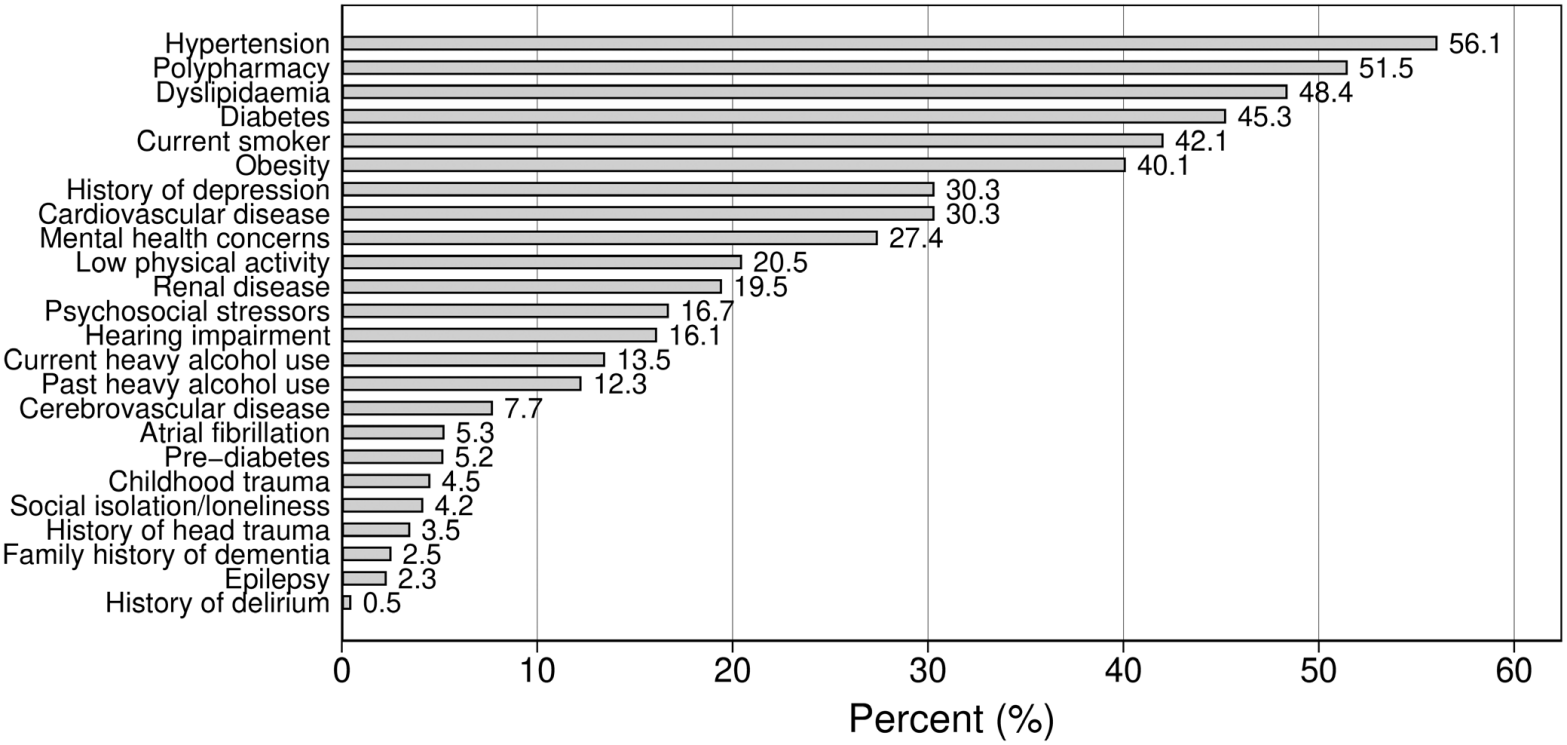
Proportion of patients with dementia‐related risk factors.

Documented dementia‐related risk factors were highly prevalent (Figure 2), particularly those associated with metabolic syndrome (hypertension, dyslipidaemia, diabetes and obesity). More than 20% had documented polypharmacy, smoking, obesity, depression, cardiovascular disease, mental health concerns, and/or low physical activity. Nearly one‐third of the sample had a history of depression (30%). Psychosocial stressors (17%), social isolation/loneliness (4%) and childhood trauma (5%) were also frequently documented.

**FIGURE 3:**
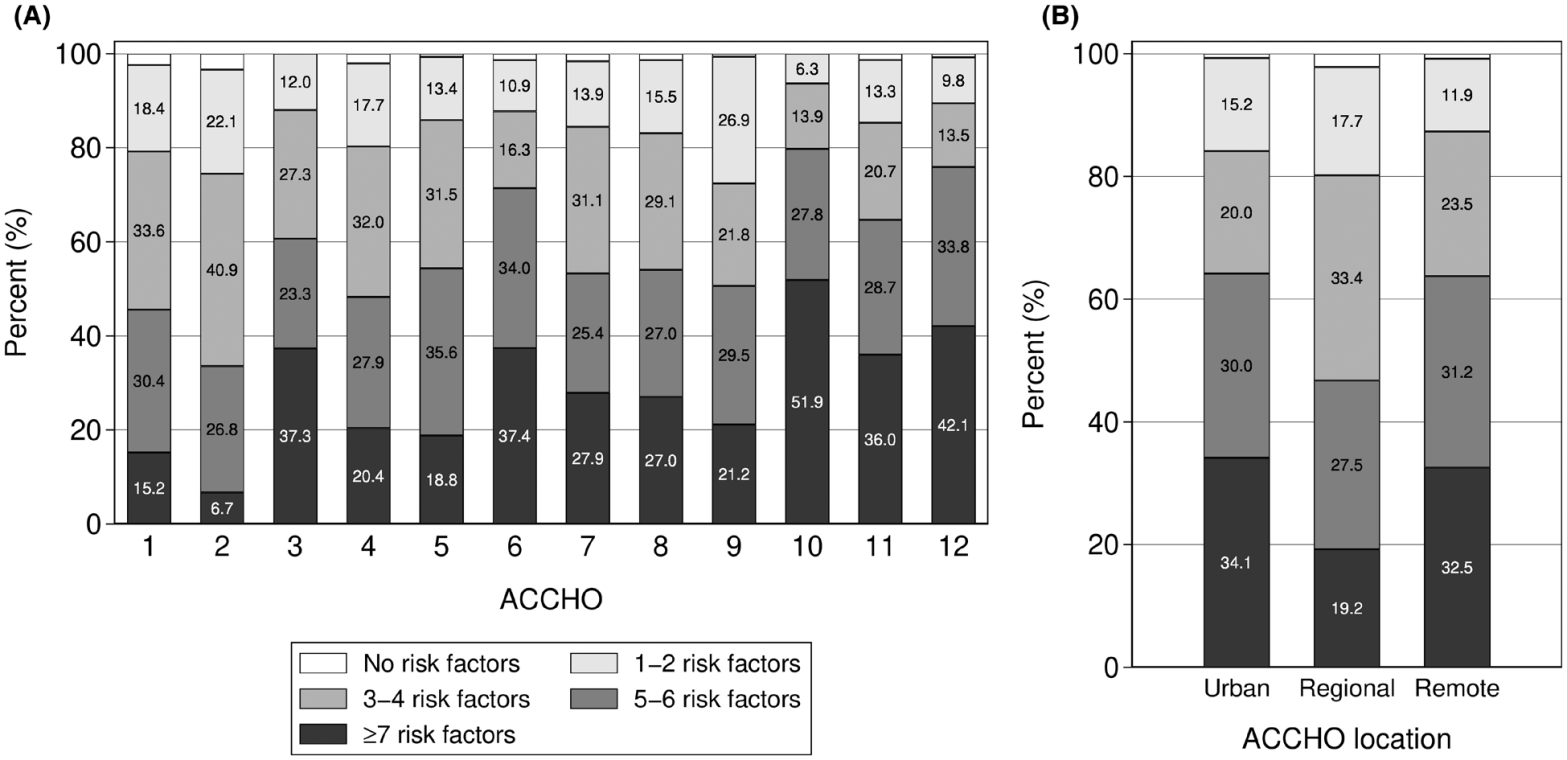
Proportion of people with dementia‐related risk factors by Aboriginal Community‐Controlled Health Organisations (ACCHO) (A) and ACCHO location (B).

The prevalence of documented dementia‐related risk factors varied considerably by ACCHO (Figure 3A). The proportion of patients within an ACCHO having ≥4 risk factors ranged from 52% to 89% (*p <* .001). For clients with ≥7 risk factors, the proportion ranged from 7% to 52% across the ACCHOs (*p <* .001). Overall, the presence of multiple risk factors was very common across the entire cohort, with over two thirds of patients (71%) documented as having ≥4 risk factors. Approximately one quarter (28%) had ≥7 risk factors. Men and women had a similar number of risk factors (*p =* .69). As shown in Figure 3B, patients at regional ACCHOs had a lower number of documented risk factors compared to their urban and remote counterparts (*p* = .049). Evidence of assessment of cognition through questions or use of assessment tools was documented for 519 patients (31%). In most cases, questions had been asked about memory, confusion and/or thinking problems (26%; *n* = 425). Formal cognitive assessment tools had been used with 209 patients (13%), the most common of which was the Mini Mental State Examination (MMSE), administered to 187 patients (11%) as shown in Table 1. Overall, the proportion of patients at each ACCHO with any documented cognitive assessment ranged from as few as 4% to as many as 84%. Evidence of cognitive assessment was noted for almost half of urban (46%) and almost one‐third of regional (30%) patients, but for only 12% of patients at remote ACCHOs. A small proportion of patients had undergone other investigations, which included laboratory investigations (4%; *n* = 68), brain imaging (5%; *n* = 76) and referral to a geriatrician, memory clinic or other specialised service (3%; *n* = 46).

Concerns about memory, confusion and/or thinking problems were documented for 158 patients (10%), including those with diagnosed/identified dementia or CIND. Concerns were raised by: a GP (41%; *n* = 64); patient (41%; *n* = 64); family member and/or carer (20%; *n* = 31); an unspecified health professional (4%; *n* = 7); an allied health professional (2%; *n* = 3); nurse (3%; *n* = 5); Aboriginal Health Worker (3%; *n* = 5); or a mental health practitioner (1%; *n* = 2).

## 4 DISCUSSION

This is the first comprehensive audit of documented cognitive impairment and associated risk factors in an Aboriginal Community‐Controlled primary health‐care setting. The audits revealed low documentation of dementia and CIND at the participating ACCHOs, demonstrating little change in rates of dementia detection over the past decade.^18^ Cognitive impairment was documented for just 4% of patients, and only 2% had a diagnosis of dementia recorded. The prevalence of detected dementia and CIND differed markedly by individual ACCHO (1%–9%); however, this did not appear to be closely related to whether they were in urban, rural or remote settings.

Levels of documented cognitive impairment were well below the known prevalence of cognitive impairment in Aboriginal and Torres Strait Islander populations, with 20%–36% diagnosed with CIND or dementia in previous dementia prevalence studies. This included in the Kimberley (12% of people aged 45 years or older with dementia and 8% with CIND),^3^ urban and rural Aboriginal communities in NSW (13% of people aged 60 years or older with dementia and 19% with CIND),^4^ and in the Torres Strait (14% of people aged 45 years or older with dementia, 22% with CIND).^5^

The results of our study illustrate the potential to improve detection of dementia and CIND with Aboriginal and Torres Strait Islander populations in primary health‐care settings. While the true prevalence of dementia and CIND may differ between ACCHOs, it is likely that contextual and/or operational differences within organisations also influence both rates of detection and patient risk factor profiles. To illustrate, one ACCHO with relatively high detection levels included cognitive questions in their Older Persons' Annual Health Check. They also had longstanding medical staff with established relationships with their patients and may have been more likely to notice cognitive changes. In contrast, an ACCHO with very low detection levels was characterised by frequent staff turnover, replacing their entire GP staff within the first 2 years of the research project. This potentially impacted the detection of CIND and dementia.

Of the 1655 patient records audited, over two‐thirds (69%) had no evidence of assessment of cognition. The low levels of both informal and formal assessment of memory and cognition, as well as the overall limited use of formal assessment tools across ACCHOs, are likely potential contributors to low detection rates of CIND and dementia.

Supporting ACCHOs to make practice changes, such as including questions about cognition in the older persons' annual health check and establishing geriatrician clinics within the ACCHOs, could be highly effective strategies to foster increased and timely detection of dementia. Implementing the updated Aboriginal and Torres Strait Islander older persons' health check templates developed by the RACGP and NACCHO, which include a section on memory and thinking, will help to encourage this practice.^24^

Documented risk factors for dementia were highly prevalent in our sample, many of which are potentially modifiable. A previous study in NSW found the prevalence of multimorbidity for Aboriginal and Torres Strait Islander patients to be 2.6 times higher than that of non‐Aboriginal patients after adjusting for socioeconomic status, age and sex,^12^ and included many dementia risk factors. It is estimated that nearly half of dementia cases could be prevented globally through the management of associated modifiable risk factors.^9^ Work examining data from health checks of Aboriginal and Torres Strait Islander people living in Far North Queensland concluded that up to 35% of the dementia burden in this population may be attributed to 11 potentially modifiable risk factors.^25^ More recently, a cross‐sectional examination of data from the National Aboriginal and Torres Strait Islander Health Survey (2018–2019) concluded that 45% of the dementia burden in the broader Australian First Nations population may be caused by 11 modifiable risk factors.^26^ Accordingly, the potential to reduce cognitive impairment and dementia through preventing and managing associated risk factors is promising.

Previous studies have highlighted the role of social determinants of health across the life course in dementia risk^6^, ^15^, ^27^ and it is clear that following a life course approach is essential in the prevention and management of dementia.^15^ While our audit tool aimed to capture risk factors connected to social determinants of health, this information was not always readily available in medical records. Increased awareness of the contribution of these factors in relation to dementia risk and the need for the management of such issues needs to be an integral component of health service delivery.

This study had several limitations. The results are based on audit data and, as a result, can only record patient information that is documented and discoverable in medical records. As such, the available data may underrepresent the true prevalence of cognitive impairment and associated risk factors in patients. Nevertheless, the observed risk factor profile was consistent with our previous Kimberley, NSW and Torres Strait studies showing a high prevalence of medical risk factors.^4^, ^17^, ^28^ The data presented are also limited by their cross‐sectional nature, and no associations between risk factors and cognitive impairment were directly examined. Longitudinal studies are needed to ascertain the relative importance of particular risk factors and clusters of risk factors and the relationship to dementia outcomes. Future research focusing on this important area and utilising culturally responsive approaches would make a valuable contribution and inform dementia prevention, detection and management approaches for Aboriginal and Torres Strait Islander peoples.

## 5 CONCLUSIONS

This study described retrospective baseline audit results of the first comprehensive audit of dementia, CIND and associated risk factors to be conducted in the Aboriginal Community‐Controlled primary health‐care setting. It forms part of ongoing programs to optimise the detection and management of cognitive impairment in older Aboriginal and Torres Strait Islander people attending primary health care, in alignment with the *Aboriginal and Torres Strait Islander Roadmap for Dementia Research and Translation*.^29^ Results demonstrated low documented levels of cognitive impairment and dementia in health‐care data, coupled with a high prevalence of associated risk factors, many of which are potentially modifiable. These findings underscore the need to improve patient care through timely and effective identification of dementia and CIND through the implementation of responsive models of cognitive care that are culturally appropriate and co‐designed with ACCHOs. They also offer a greater understanding of risk factors in terms of the prevalence of different risk factors and the co‐occurrence of risk factors within individuals. This is valuable information that can be used to guide prevention strategies and risk factor management initiatives going forward.^30^

## FUNDING INFORMATION

This project is funded by two National Health and Medical Research Council (NHMRC) grants: 1137425 and 1150337. The funder had no role in this study.

## CONFLICT OF INTEREST STATEMENT

No conflicts of interest declared.

## Supporting information


Appendix S1

## Data Availability

The audit tool is available as Appendix S1. The data that support the findings of this study are available on request from the corresponding author. The data are not publicly available due to privacy or ethical restrictions.
