# Supplementary material for: Detection of cognitive impairment, dementia and associated risk factors among Aboriginal and Torres Strait Islander peoples: Retrospective baseline audit results from a stepped‐wedge cluster‐randomised controlled trial
Source: Australas J Ageing. 2025 Mar 5;44(1):e70007. doi: 10.1111/ajag.70007 (PMC11882482; doi:10.1111/ajag.70007)
Supplement: Supplementary file 1 — Appendix S1 [file AJAG-44-0-s001.pdf]

Lets Chat Dementia Audit Tool

Client ID	__________________
Date of audit	__________________
Client attendance status	◯ Client HAS attended the clinic during the six month audit period<br>◯ Client has NOT attended the clinic during the six month audit period<br>◯ Client has been moved into residential care<br>◯ Client is no longer using the clinic, and has either moved away or is attending another clinic<br>◯ Client is deceased<br>◯ Unsearchable in Medical Record database<br>◯ Lost to follow up<br>◯ Patient has become inactive in medical record database<br>(Ensure that attendance status is updated for each audit period.)
Client age on last day of audit period	__________________
Date deceased	__________________
Usual living arrangements	◯ Lives alone<br>◯ Lives with partner/family<br>◯ Lives in residential care<br>◯ Other - specify<br>◯ Not specified
Living arrangement other - specify	__________________
Does the client live in more than one place for extended periods?	◯ Yes<br>◯ Sometimes<br>◯ No<br>◯ Not Specified
Is overcrowding an issue for this client in their place of residence?	◯ Yes<br>◯ No<br>◯ Not Specified
Is the client at risk of homelessness?	◯ Yes<br>◯ No<br>◯ Not specified
Is the client homeless?	◯ Yes<br>◯ No<br>◯ Not specified

Is the client a carer?	◯ Yes<br>◯ No<br>◯ not specified
Cares for?	☐ Spouse/partner<br>☐ Parent<br>☐ Daughter/son<br>☐ Granddaughter/grandson<br>☐ Other family member<br>☐ Other - specify
Cares for other - specify	______________________________
Does this client have an informal carer(s)?	◯ Yes<br>◯ No<br>◯ Not specified
Who is/are the client's informal carer(s)?	☐ Spouse/partner<br>☐ Parent<br>☐ Daughter/son<br>☐ Granddaughter/grandson<br>☐ Other family member<br>☐ Other
Other type of carer - specify	______________________________
Highest level of education attained	◯ no formal education/ some primary school<br>◯ completed primary school<br>◯ some secondary education completed (eg. to year 9/10/11)<br>◯ completed secondary school<br>◯ TAFE certificate<br>◯ tertiary education / university degree<br>◯ Other<br>◯ Not specified
Other education - specify	______________________________
Client's employment status?	◯ Employed part time<br>◯ Employed full time<br>◯ Unemployed<br>◯ Retired<br>◯ Disability pension<br>◯ Old age pension<br>◯ Unknown
Specify further details (as needed)	______________________________

Have any of the following MBS Item numbers been claimed since the previous audit (for 1st audit in past 2 years)?	☐ 715 (Health Assessment), 92004, 92016 (telehealth/telephone)<br>☐ 721 (GPMP, Care plan), 92024, 92068 (telehealth/telephone) / 732 (Care plan review), 92028, 92072 (telehealth/telephone)<br>☐ 723 (TCA) Team Care Arrrangement, 92025, 92069 (telehealth/telephone)<br>☐ 900 (DMMR/HMR) Medication Review<br>☐ 935-958 (Care Team Meeting/Case Conferencing)<br>☐ 2700-2717 (GP Mental Health Treatment Plan), 92112-92117, 92124-92129 (telehealth/telephone)<br>☐ 10987-10989, 10991,10997 (AHW/P or nurse), 93200 -93202 (telehealth/telephone)<br>☐ 82200-82215 (Nurse practitioner), 91192, 91178 -91180, 91193, 91189-91191 (telehealth/telephone)<br>☐ 10950, 81300 (AHW/P), 93000, 93048, 93013, 93061 (telehealth/telephone)<br>☐ 10951-10970 (Allied health), 9300, 93013 (telehealth/telephone)
Is the client enrolled in My Aged Care?	◯ yes<br>◯ no<br>◯ not specified
Risk factors for dementia documented.	☐ Smoking status recorded<br>☐ Diabetes<br>☐ Cardiovascular disease (refer to glossary)<br>☐ Cerebrovascular disease (stroke, TIA, CVA)<br>☐ Atrial fibrillation (AF, AFib)<br>☐ History of delirium<br>☐ Epilepsy<br>☐ Hearing impairment<br>☐ Obesity<br>☐ Heavy alcohol use -past<br>☐ Heavy alcohol use - current<br>☐ History of head trauma<br>☐ History of depression<br>☐ Significant psychosocial stressors<br>☐ History of childhood trauma<br>☐ Polypharmacy (5 or more medications)<br>☐ Social isolation/loneliness<br>☐ Low physical activity<br>☐ Family history of dementia<br>☐ No risk factors documented<br>☐ Pre-diabetes<br>☐ Dyslipidaemia, hyperlipidaemia, hypercholesterolaemia, high cholesterol<br>☐ Hypertension<br>☐ Renal disease (Kidney disease, CKD, ESRD)<br>☐ Documentation of mental health concerns
Smoking status	◯ Current smoker<br>◯ Ex-smoker<br>◯ Never smoked
Have concerns about memory, confusion or thinking problems been raised by/in relation to this client, in this/or any prior audit periods?	◯ Yes<br>◯ No

In which audit period(s) have concerns been raised?	☐ Audit 1,<br>☐ Audit 2,<br>☐ Audit 3,<br>☐ Audit 4,<br>☐ Audit 5,<br>☐ Audit 6,<br>☐ Audit 7,<br>☐ Audit 8,
Who raised concerns?	☐ Client<br>☐ Family member/carer<br>☐ Health professional (type not specified)<br>☐ AHW<br>☐ Nurse<br>☐ GP<br>☐ Allied health<br>☐ Mental health practitioner (psychologist, social worker, therapist, AOD worker)<br>☐ Not specified<br>☐ Other
Other - specify	__________________________________
Evidence of assessment of cognition within the current audit period (NOTE: For Audit 1 only, check entire patient history).	☐ Questions about memory, confusion or thinking problems<br>☐ Cognitive assessment tools<br>☐ No evidence<br>(Only look back in the client history for audit 1. For all subsequent audits only check within the audit period. E.g only mark Questions about memory. confusion or thinking and/or cognitive assessment tools for audit 2 if the person has had this done in the audit 2 period.)
Who asked questions about memory, confusion or thinking?	☐ Primary Care Team (GP, RN, AHW/P, allied health, mental health)<br>☐ GP<br>☐ RN<br>☐ AHW/P<br>☐ Allied health<br>☐ Mental health (within service)<br>☐ Mental health (external to service)<br>☐ Geriatrician/other specialist<br>☐ ACAT assessor or team<br>☐ Other
Specify type of allied health professional	__________________________________
What tools were used to assess cognition?	☐ MMSE<br>☐ KICA<br>☐ Clock test<br>☐ GP-Cog<br>☐ RUDAS<br>☐ Other
Specify which other tool(s) were used	__________________________________

Cognitive assessment(s) results/comments

Who conducted the cognitive assessment(s)?

- ☐ Primary Care Team (GP, RN, AHW/P, allied health, mental health)
- ☐ GP
- ☐ RN
- ☐ AHW/P
- ☐ Allied health
- ☐ Mental health (within service)
- ☐ Mental health (external to service)
- ☐ Geriatrician/other specialist
- ☐ ACAT assessor or team
- ☐ Other

Specify type of allied health professional

____

Evidence of assessment of decision-making capacity within the current audit period (NOTE: For Audit 1 only, check entire patient history).

- ☐ Comments about insight, reasoning ability, decision-making
- ☐ No evidence

(Only look back in the client history for audit 1. For all subsequent audits only check within the date range of the audit period. E.g only mark comments about insight, reasoning, decision -making for audit 2 if the person has had this done in the audit 2 period.)

Evidence of investigation of cognitive impairment within the current audit period (NOTE: For Audit 1 only, check entire patient history).

- ☐ Documentation of laboratory investigations relating to CI/dementia
- ☐ CT-Brain or MRI-Brain
- ☐ No evidence

(Only look back in client history for audit 1. For all subsequent audits only check within the date range of the audit period. E.g. you would only mark CT-Brain or MRI-Brain for audit 2 if the person has had this done in the audit 2 period.)

What was the clinical indication for CT or MRI?

____

Has CI been identified in this or previous audit periods?

- ◯ Yes
- ◯ No

In which audit period(s) has CI been identified?

- ☐ Audit 1,
- ☐ Audit 2,
- ☐ Audit 3,
- ☐ Audit 4,
- ☐ Audit 5,
- ☐ Audit 6,
- ☐ Audit 7,
- ☐ Audit 8,

What diagnosis relating to CI has been identified?	☐ Dementia<br>☐ MCI<br>☐ Depression<br>☐ Delirium<br>☐ Medication-related<br>☐ Head trauma/ABI (chronic traumatic encephalopathy (CTE))<br>☐ No diagnosis<br>☐ Other
Other (specify)	__________________
Type of dementia diagnosed	☐ Alzheimer's<br>☐ Vascular<br>☐ Mixed Dementia<br>☐ Lewy Body<br>☐ Parkinson's<br>☐ Frontotemporal<br>☐ Younger Onset Dementia<br>☐ Not specified
Date of diagnosis of MCI/D	__________________

19.1 Standard care for clients with CI/D - medical

	Documented	Not applicable
Management of current smoking	☐	☐
Management of current alcohol consumption	☐	☐

20.1 Standard care for clients with CI/D - risk assessments

	Assessed	Risk identified	Evidence of health service response	Not applicable
Falls	☐	☐	☐	☐
Pain	☐	☐	☐	☐
Delirium	☐	☐	☐	☐
Continence	☐	☐	☐	☐
Nutrition	☐	☐	☐	☐
Elder abuse considered	☐	☐	☐	☐

21.1 Standard care for clients with CI/D - functional assessments

	Assessed	Unknown	Not applicable
Activities of daily life (ADLs)	○	○	○
Handling finances	○	○	○
Driving	○	○	○

Who conducted the functional assessment(s)?

- ☐ Primary care team (GP, RN, AHW/P, Allied health)
- ☐ GP
- ☐ RN
- ☐ AHW/P
- ☐ Allied health
- ☐ Geriatrician/other specialist
- ☐ ACAT assessor or team

22.1 Assessment/management of neuropsychiatric symptoms

	Assessed	Identified	Evidence of response from health service	Not applicable
General mental health (Depression, Anxiety, Agitation)	☐	☐	☐	☐
Depression	☐	☐	☐	☐
Anxiety	☐	☐	☐	☐
Agitation	☐	☐	☐	☐
Sleep disturbance	☐	☐	☐	☐
Psychosis	☐	☐	☐	☐
Other BPSD (eg. wandering, aggression, disinhibition)	☐	☐	☐	☐

Model of care

- ☐ Medication review (MBS 900 - DMMR, HMR)
- ☐ Current care plan (MBS 721) or review of care plan (MBS 732) in previous six months
- ☐ Referral to allied health (TCA MBS 723, physio, OT, podiatry, other)
- ☐ Care team/case conferencing meetings (MBS 735-758)
- ☐ Referral to geriatrician/memory clinic/other specialist service
- ☐ Client referral to support services
- ☐ Carer referral to support services
- ☐ ACAT/NSAF assessment completed
- ☐ Family meetings
- ☐ Dementia medication prescribed

Provider of medication review

- ☐ GP
- ☐ Pharmacist & GP (DMMR/HMR MBS 900)

Allied health - specify

- ☐ TCA (MBS 723)
- ☐ Physio
- ☐ OT
- ☐ Podiatry
- ☐ Other

Other allied health - specify

____

Support services being used by client

- ☐ Respite
- ☐ Transport
- ☐ ADLs/home visits (eg. cooking, shopping, showering, housework, medicine management)
- ☐ Social connection (eg. day programs, outings, cultural events)
- ☐ Other

Other support services used by client (specify)	__________________________
Support services being used by carer	☐ Respite<br>☐ Counselling<br>☐ Social connection (eg. carer support group)<br>☐ Other
Other support services used by carer (specify)	__________________________
Which dementia medications have been prescribed?	☐ Donepezil: APO donepezil, Arazil, Aridon, Aricept<br>☐ Galantamine: Gamine, Reminyl<br>☐ Rivastigmine: Exelon, Rivastigmelon<br>☐ Memantine: Ebixa, Memanxa
Proxy decision maker (power of attorney) appointed	◯ yes<br>◯ no<br>◯ not specified
Future/end-of-life (EOL) planning (eg. consideration of return to Country, ACP)	◯ yes<br>◯ no<br>◯ not specified
Evidence of consideration of carer health and wellbeing	◯ Yes<br>◯ No
Details of carer well-being inquiry	__________________________
Provision of general primary health care	☐ Evidence of discussion re healthy lifestyle (diet, exercise, etc.)<br>☐ Dental care<br>☐ Immunisation<br>☐ Other (eg. cancer screening)
Has the client been hospitalised in the past two years (first audit only) or since the previous audit?	◯ Yes<br>◯ No
Please record the number of hospital admissions in the past two years (1st audit only) or since the previous audit.	__________________________
Reason(s) for hospitalisation(s)	__________________________
Additional comments (eg. education, employment, other relevant info)	
